# AGIG Chemo-Immunotherapy in Patients With Advanced Pancreatic Cancer: A Single-Arm, Single-Center, Phase 2 Study

**DOI:** 10.3389/fonc.2021.693386

**Published:** 2021-10-13

**Authors:** Wangshu Dai, Xin Qiu, Changchang Lu, Zhengyun Zou, Huizi Sha, Weiwei Kong, Baorui Liu, Juan Du

**Affiliations:** ^1^ The Comprehensive Cancer Center of Drum Tower Hospital, Medical School of Nanjing University & Clinical Cancer Institute of Nanjing University, Nanjing, China; ^2^ The Cadre Health Care Ward, Nanjing Drum Tower Hospital, The Affiliated Hospital of Nanjing University Medical School, Nanjing, China; ^3^ The Comprehensive Cancer Center of Drum Tower Hospital, Clinical College of Traditional Chinese and Western Medicine, Nanjing University of Chinese Medicine, Nanjing, China

**Keywords:** objective response rate, overall survival, advanced pancreatic adenocarcinoma, chemo-immunotherapy, nab-paclitaxel, gemcitabine

## Abstract

**Background:**

To date, chemotherapy remains the only effective treatment of unresectable pancreatic adenocarcinoma. In the past few years, the interest in immunological anticancer therapy rises sharply. AGIG is a novel chemo-immunotherapy regimen that combines nab-paclitaxel + gemcitabine chemotherapy with sequential recombinant interleukin-2 (IL-2) and granulocyte-macrophage colony stimulating factor (GM-CSF) therapy. We conducted a single-arm prospective phase II study to determine the efficacy and safety of the first-line treatment of advanced pancreatic cancer with AGIG regimen.

**Methods:**

Nab-paclitaxel (125 mg/m^2^) and gemcitabine (1000 mg/m^2^) were administered intravenously to all patients on days 1 and 8 triweekly, interleukin-2 (1000000U) and GM-CSF (100 µg) were administered subcutaneously on days 3-5 after chemotherapy. The primary end point was ORR by the Response Evaluation Criteria in Solid Tumors, version 1.1. Secondary end points included safety profile, progression-free survival (PFS), overall survival (OS). Patients’ conditions along with the efficacy and safety were assessed every two cycles.

**Results:**

Between 11/2018 and 01/2020, sixty-four patients were enrolled. In the sixty-four evaluable patients, the disease control rate (DCR) and overall response rate (ORR) were 76.6% and 43.75%, respectively. The median follow-up time was 12.1 (range 7.1–22.4) months. The median PFS was 5.7 (range 1.63–15.8) months. The median OS was 14.2 (range 2.9–22.0) months. The most common adverse event was fever (75%). The incidence of III/IV grade neutropenia was 4.69%. In subgroup analyses, we found that eosinophil count in the blood elevated three times higher than baseline level predicted a longer survival.

**Conclusions:**

The AGIG chemo-immunotherapy regimen has presented favorable ORR, OS, and manageable toxicities as first-line therapeutic strategy of advanced pancreatic cancer treatment. This regimen may be a novel reliable therapeutic option for patients with preserved performance status. The improvement of treatment efficiency may be related to the activation of non-specific immune response.

**Clinical Trial Registration:**

https://clinicaltrials.gov/. identifier NCT03768687.

## Introduction

Pancreatic cancer is one of the deadliest solid malignancies in the world. Despite decades of efforts, it remains the fourth leading cause of cancer-related death worldwide, with a five-year survival rate of less than 5% (1). Without treatment, the median survival time is consistently shorter than six months (2). Since 1997, gemcitabine had been the standard treatment for unresectable pancreatic adenocarcinoma (3). After decades of exploration, both FOLFIRINOX (fluorouracil, irinotecan, and oxaliplatin) and nab-paclitaxel with gemcitabine (AG) prolong overall survival (OS) compared with gemcitabine alone (4, 5). Till now, chemotherapy remains the only effective treatment of unresectable pancreatic adenocarcinoma (6).

As mentioned above, FOLFIRINOX is one of the standard treatment strategies for patients with advanced pancreatic cancer and has demonstrated good effectiveness in Europe and North America (7, 8). However, grade III/IV adverse events were commonly observed in the FOLFIRINOX treatment courses. To our knowledge, cancer drug tolerability is different between Asian and white populations. These differences may be related to genetic or environmental factor. Increased chemotherapy-induced myelo-suppression was one of the most commonly observed adverse events in Asian patients (9, 10). Chinese patients were unendurable to FOLFIRINOX chemotherapy sometimes. The combination of nab-paclitaxel and gemcitabine is recommended as the first-line treatment regimen for patients with advanced pancreatic cancer by the National Comprehensive Cancer Network (NCCN) guidelines. A phase I/II study evaluated the AG chemotherapy regimen in Chinese patients with advanced pancreatic cancer (11). The study was carried out at a dose and schedule different from the classic MPACT study. The recommended administration schedule was described as follows, nab-paclitaxel (125 mg/m^2^) along with gemcitabine (1000 mg/m^2^) was administered on the first day and the eighth day, the treatment was repeated every three weeks (12). Although the trail did not meet its primary endpoint of identifying the maximum tolerated dose in Chinese pancreatic cancer sufferers, the study showed a manageable safety profile with a favorable antitumor effect in pancreatic cancer sufferers.

With the clinical development and application of PD-1/PD-L1 immune-checkpoint blockade, the interest in the exploration of immunological anticancer strategies rises sharply in these years. Immune-based regimens are showing promise where other approaches have failed when treating pancreatic cancer (13, 14). Immune checkpoint inhibitors along with therapeutic vaccines and combination immunotherapies are commonly used as immunotherapeutic strategies. Even though the antitumor effect and mechanism of the above-mentioned immunotherapeutic strategies remain unclear, these researches produced abundant data concerning the mechanisms of the efficient tumor-specific adaptive immune response triggered by immune-modulating agents (15).

It was reported that chemo-immunotherapy might represent as an innovative reliable therapy option for first-line treatment of metastatic colorectal cancer sufferers (16, 17). Interleukin-2 (IL-2) was used to promote the proliferation of cross-primed cytotoxic T lymphocyte clones, while granulocyte-macrophage colony stimulating factor (GM-CSF) was required to activate the antigen-presenting ability of the dendritic cells expressed in human peripheral blood mononuclear cells. GM-CSF is essential for the differentiation of dendritic cells, which are responsible for processing and presenting tumor antigens for the priming of antitumor cytotoxic T lymphocytes (18, 19). Some GM-CSF-based cancer immunotherapy strategies have been developed for in clinical practice (20). It was reported that IL-2 and GM-CSF were demonstrated as innovative and reliable adjuvants of chemotherapy for metastatic colorectal cancer (21, 22). These results offered the rationale to design a novel treatment chemo-immunotherapy regimen that combines traditional chemotherapy with IL-2 and GM-CSF.

AGIG is a novel chemo-immunotherapy regimen that combines AG chemotherapy with sequential recombinant IL-2 and GM-CSF therapy (nab-paclitaxel, gemcitabine, IL-2 and GM-CSF). In this study, we implemented a single-arm, single-center prospective phase II study to determine the efficacy and safety of the AGIG regimen as the first-line treatment of advanced pancreatic cancer in China.

## Materials and Method

### Patients

This was a prospective study involving pancreatic sufferers receiving AGIG Chemo-immunotherapy regimen from November 2018 to January 2020 at the Comprehensive Cancer Centre of Drum Tower Hospital, Clinical Cancer Institute of Nanjing University. In all cases, a multidisciplinary team participated in the diagnosis of pancreatic adenocarcinoma followed by the NCCN guidelines. Patients with Eastern Cooperative Oncology Group (ECOG) performance score higher than 1, inadequate bone marrow, abnormal liver or renal functions, additional other malignancies, and patients older than eighty-five years were excluded. Patients enrolled in the trial were prescribed AGIG regimen.

### Procedures

**Figure 1:**
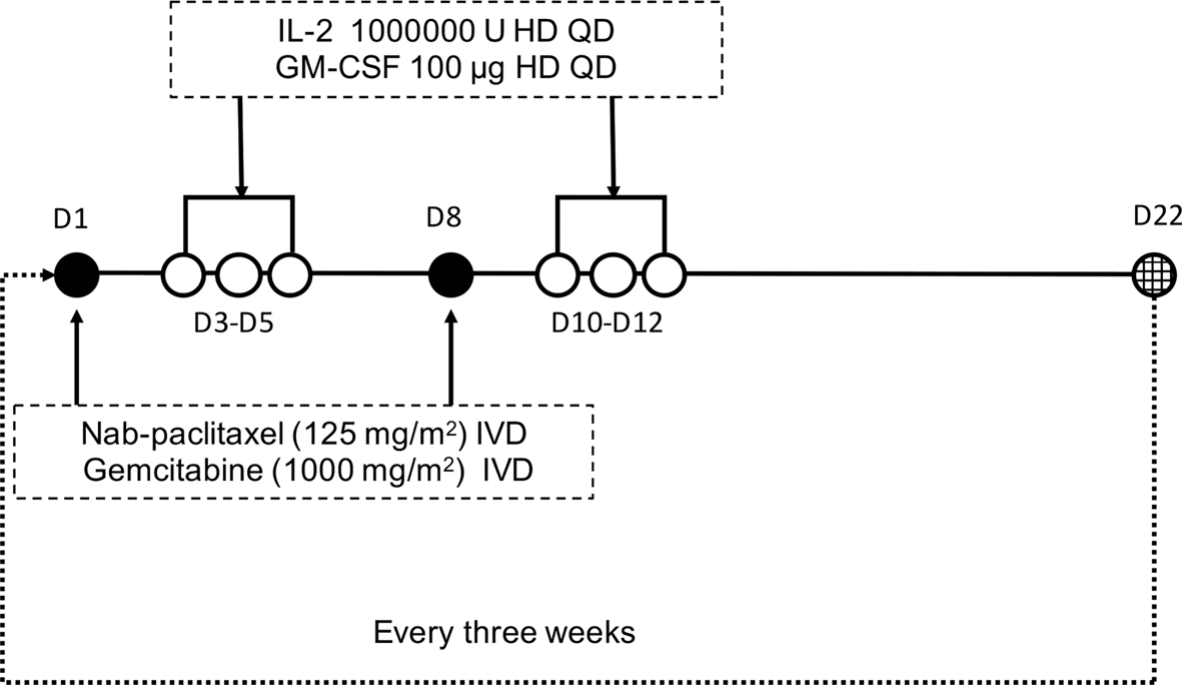
Protocol of drug administration. Nab-paclitaxel (125 mg/m^2^) and gemcitabine (1000 mg/m^2^) were administered intravenously to all patients on days 1 and 8 triweekly. Interleukin-2 (10000000 U) and GM-CSF (100 µg) were administered subcutaneously on days 3-5 after chemotherapy.

Patients enrolled in the trial received AGIG chemo-immunotherapy. Nab-paclitaxel (125 mg/m2) and gemcitabine (1000 mg/m2) were administered intravenously to all patients on the first day and the eighth day of the treatment cycle. IL-2 (10000000 U) and GM-CSF (100 µg) were administered subcutaneously on three to five days after chemotherapy. The treatment is repeated every three weeks. **Figure 1** showed the drug administration protocol of the AGIG regimen. We evaluate clinical and laboratory results at baseline and repeated every time before chemotherapy. Radiographic response evaluation was performed every six weeks. Subjects continued their treatment until disease progression, clinical judgment, occurrence of unacceptable toxicity, or withdrawal of consent. Supportive care was permitted during the treatment course. Second line therapy after disease progression was left to the discretion of the treating oncologist.

### Assessment

All patients were evaluated every two cycles of AGIG chemo-immunotherapy using multislice computed tomography scans with contrast medium. Physical examination and laboratory tests including blood routine test, biochemical index and serum CA199 assays were performed every time before chemotherapy. We categorize tumor response into complete response (CR), partial response (PR), stable disease (SD), and progressive disease (PD) according to the Response Evaluation Criteria in Solid Tumors (version 1.1). With respect to the safety observation of the treatment, we graded adverse events according to the National Cancer Institute Common Terminology Criteria for Adverse Events (version 4.0). Overall survival (OS) was defined as the duration from the beginning of chemotherapy to the date of death of any cause. Progression free survival (PFS) was defined as the duration from the beginning of chemotherapy to the date of disease progression or death. Subjects without event were censored at the last follow-up date (August 1st, 2020). Characteristic files were collected at the moment of admission.

### Statistical Analysis

All data were analyzed using Graphpad Prism 6 and SPSS software (version 21.0). Survival analyses were performed using the Kaplane-Meier method, Log-rank (Mantel-Cox) tests and Gehan-Breslow-Wilcoxon tests. Data were presented as median and range. A P value less than 0.05 was considered statistical significance.

## Results

### Patient Characteristics

**Figure 2:**
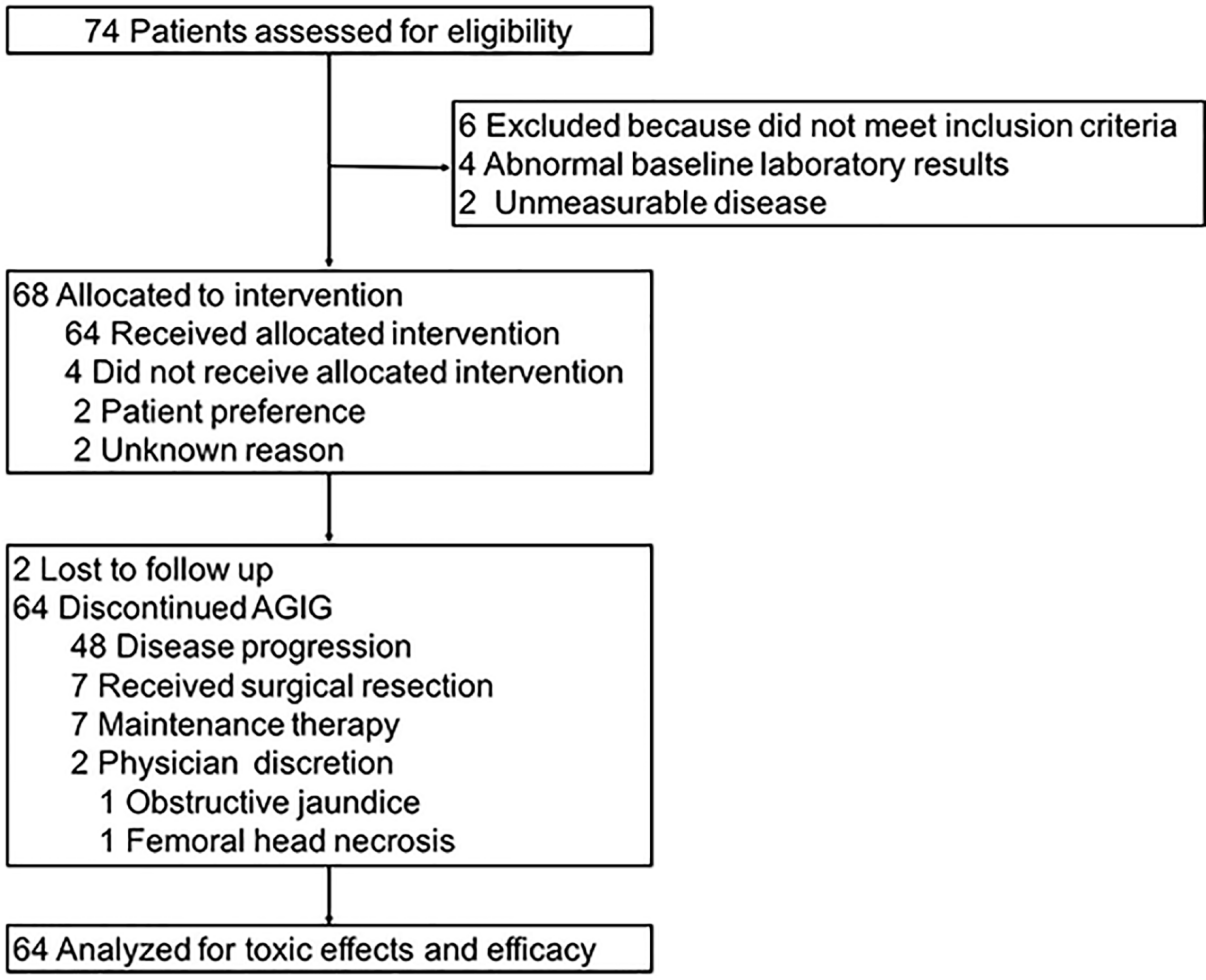
Study flowchart.

Between 11/2018 and 01/2020, a total of sixty-four patients were enrolled and evaluated in our trial. **Figure 2** showed the study flowchart of this trial. Patient characteristic files at baseline are summarized in **Table 1**. There were thirty-six (56.25%) males and twenty-eight (43.75%) females. The median age was 62 (range 33 - 81) years. All subjects were ECOG PS 0–1. Fifty-nine PC sufferers (92.19%) had elevated baseline CA199, with a median value of 1033 (range 27 - 30491) u/mL. In total, 51.56% (n = 33) of the tumors were located in the head and neck of the pancreas. 40.63% (n = 26) of the tumors were located in the body or tail of the pancreas. 49 patients were histologically diagnosed as adenocarcinoma including one case of cystadenocarcinoma and one case of mucinous adenocarcinoma. 4 patients were histologically diagnosed as adeno-squamous carcinoma, and none of the patients developed undifferentiated or undifferentiated neuroendocrine carcinoma. In addition, 11 patients’ pathological types are still unknown due to the limited pathologic sampling ability of endoscopic ultrasonography. Nearly half of enrolled patients (n = 31, 48.44%) had metastases at the initial diagnosis. The majority of the cases had liver (n = 23, 74.19%) or peritoneal (n = 10, 32.26%) metastasis. Seven patients had undergone a prior resection.

**Table 1:** Baseline characteristics. (intention-to-treat population).

Characteristic	AGIG (N = 64)	No. (%)
Sex		
Male	36	56.25%
Female	28	43.75%
Age, median (range),y	62 (33-81)	
ECOG PS		
0	19	29.70%
1	45	70.30%
CA 19-9 level at baseline		
median (range), u/ml	1033 (27-30491)	
<37 × ULN Normal	28	43.75%
≥37 × ULN Normal	31	48.43%
Unknown	5	7.80%
Tumor site		
Head and neck	33	51.56%
Body and tail	26	40.63%
Unknown	5	7.81%
Histology		
Adenocarcinoma	49	76.56%
Adeno-squamous carcinoma	4	6.25%
Unclear	11	17.19%
Stage		
Resection	7	10.94%
locally advanced	26	40.63%
Metastatic	31	48.44%
Site of metastatic disease	N = 31	No.(%)
Liver	23	74.19%
Lung	4	12.90%
Peritoneum	10	32.26%
Bone	2	6.45%
No. of metastatic sites		
1	1	3.23%
2	5	16.13%
3	1	3.23%
>3	24	77.25%

### Treatment Completion Rates

Seventy-four subjects were assessed for eligibility initially. Six patients were excluded because they did not meet inclusion criteria. Four patients had abnormal baseline laboratory results and two patients had immeasurable disease. Sixty-eight patients were allocated to intervention. Four patients did not receive allocated intervention, two for patient preference and two for unknown reason. A total of sixty-four patients proceeded to AGIG and were analyzed for toxic effects and efficacy. Forty-eight patients were observed disease progression throughout the follow up. Seven patients received surgical resection. Seven patients had been receiving maintenance therapy till the last follow-up date (August 1st, 2020). Two patients drop out the trial, 1 with obstructive jaundice and 1 with femoral head necrosis.

### Radiographic Response Evaluation

Radiographic response was measured with RECIST 1.1 every two cycles of AGIG chemo-immunotherapy. With sixty-four patients evaluated, two patients discontinued chemotherapy early because of obstructive jaundice and femoral head necrosis. Twenty-eight patients (43.75%) had PR, twenty-one patients (32.81%) had SD, and fifteen patients (23.43%) had PD. **Table 2** summarized the detail information of best response. For all patients (n = 64), the overall response rate (ORR) and disease control rate (DCR) was 43.75% and 76.6% respectively. No significant difference in treatment response rate was observed between the two primary tumor sites.

**Table 2:** Response rate by treatment group.

Best Response	Patients No. (%) Overall (n = 64)	Patients No. (%)		
		Head and neck	Body and tail	NA
Partial response	28 (43.75)	15 (45.45)	10 (38.46)	3 (6)
Stable disease	21 (32.81)	10 (30.30)	10 (38.46)	1 (20)
Progressive disease	15 (23.43)	8 (24.24)	6 (23.08)	1 (20)
Disease control rate	49 (76.56)	25 (75.76)	20 (76.92)	4 (80)

### Survival Analysis and Subgroup Analysis

**Figure 3:**
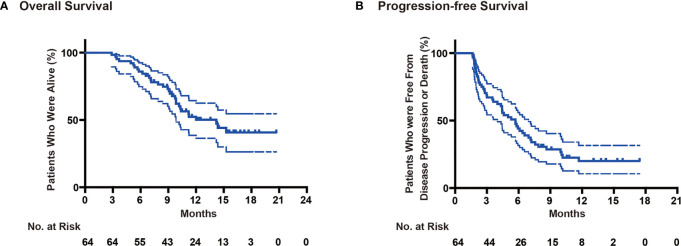
Survival analysis. **(A)** Overall survival, **(B)** Progression-free survival.

**Figure 4:**
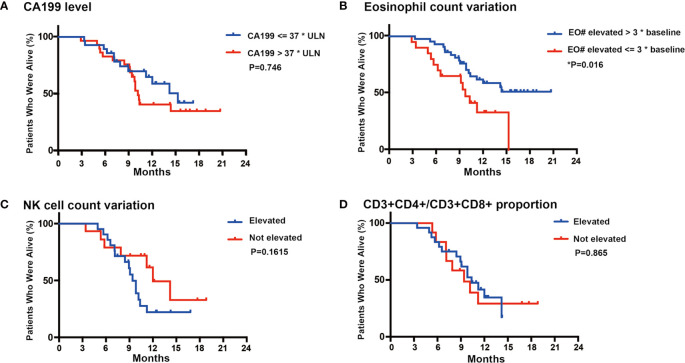
Subgroup analysis in CA199 level **(A)**, Eosinophil count variation **(B)**, NK cell count variation **(C)** and CD3+CD4/CD3+CD8+ proportion **(D)**. *P < 0.05.

At the last follow-up (1st August 2020), thirty-two patients (50%) had died. All sixty-four patients were included for survival analysis. The median follow-up time was 12.1 (range 7.1–22.4) months. For all patients, the median PFS was 5.7 (range 1.63–15.8) months (**Figure 3A**), the median OS was 14.2 (range 2.9–22.0) months (**Figure 3B**), and the one-year survival rate was 65%. We performed subgroup survival analyses in CA199 level (**Figure 4A**), eosinophil count variation (**Figure 4B**), NK cell count variation (**Figure 4C**) and CD3+CD4/CD3+CD8+ proportion (**Figure 4D**). We found that eosinophil count in the blood elevated three times higher than baseline level predicted a longer survival (P = 0.016) (**Figure 4B**).

### Adverse Events

The therapy-related toxicities are summarized in **Table 3**. Generally, the incidence of adverse events was 79.69% (n=51), and fever was the most common side effect, with an incidence of 75%. For severe adverse events, thirty-eight patients (59.38%) were observed with grade III/IV toxicities, among which 84.21% (n = 32) was alopecia, 26.32% (n = 10) was peripheral sensory neuropathy, 7.89% (n = 3) was neutropenia, 13.16% (n = 5) was thrombocytopenia, 10.53% (n = 4) was anemia. Totally, three patients (4.69%) received granulocyte-colony stimulating factor treatment before or after chemotherapy. In addition, five patients (3.2%) were treated with thrombopoietin. No patients suffered adverse event leading to death.

**Table 3:** Summary of adverse events.

Adverse event	Any grade (n = 64)	Grade 3-4 (n = 64)
White blood cell decreased	25 (39.06%)	3 (4.69%)
Anemia	33 (51.56%)	4 (6.25%)
Platelet count decreased	20 (31.25%)	5 (7.81%)
Neutrophil count decreased	32 (50%)	3 (4.69%)
Diarrhea	2 (3.13%)	0 (0%)
Rash maculopapular	22 (34.38%)	5 (7.81%)
Alopecia	52 (81.25%)	32 (50%)
Fatigue	28 (43.75%)	9 (14.06%)
Fever	48 (75%)	6 (9.38%)
Nausea	42 (65.63%)	12 (18.75%)
Vomiting	23 (35.94%)	15 (23.44%)
Dysgeusia	20 (31.25%)	0 (0%)
Anorexia	32 (50%)	8 (12.5%)
Peripheral sensory neuropathy	26 (40.6%)	10 (15.6%)
Adverse event leading to death	0 (0%)	

## Discussion

Generally, advanced pancreatic ductal adenocarcinoma is considered an incurable presentation of PC. This trial was carried out to investigate the efficacy and safety of the AGIG regimen in Chinese patients with advanced PC. In the present study, the ORR was 43.75%, with a significantly higher ORR compared with MPACT study (22.96%, n=431) (p =0.0007) (12) and a slightly higher ORR compared with LAPACT study (33.64%, n=107) (23) and HALO 202 study (32.60% n=92) (3). Other efficacy endpoints (PFS, 5.7 months; OS, 14.2 months) were not inferior to the findings of the MPACT study (PFS, 5.5 months; OS, 8.5 months) (12) and the study of Karasic et al. (PFS, 6.4 months; OS, 12.1 months) (24). The PFS was a little shorter in this study than that in the study of Karasic et al. (5.7 months vs 5.5 months). We hypothesize that these discrepancies are attributed to differences in the radiographic response measuring frequency, with biweekly measurement in this study versus triweekly measurement in the study of Karasic et al. (24). The effectiveness of the AGIG regimen was also favorable compared to other first-line treatment options presented by previous studies in patients with advanced PC (25–27). Accordingly, we consider the AGIG regimen to be not inferior to the traditional therapy regimen in Chinese patients with advanced PC.

The toxic effects of AGIG were modest. Two patients discontinued chemotherapy early because of obstructive jaundice and femoral head necrosis and none of the patients required discontinuation of AGIG because of toxicity. Exhilaratingly, we observed an obviously decrease in incidences of neutropenia and thrombocytopenia. Totally, only eight patients (12.5%) received granulocyte-colony stimulating factor (3/8) or thrombopoietin (5/8) treatment before or after chemotherapy. We attribute the results to the application of GM-CSF. GM-CSF is an important hematopoietic growth factor and immune modulator. It stimulates the proliferation of macrophage, granulocyte, erythroid, eosinophil, megakaryocyte and multipotent progenitors cells depending on its concentration (28). It also controls eosinophil function in some cases (29, 30). Fever was the most common adverse effect in sequential administration period of interleukin-2 and GM-CSF. Rash maculopapular, alopecia, fatigue, nausea, vomiting, peripheral neuropathy, and neuropsychiatric symptoms were seen with AGIG regimen. However, these effects did not lead to decreased chemotherapy intensity or treatment discontinuation. These findings suggest that lower incidence of myelosuppression in AGIG regimen ensured full dose of drug administration and sufficient course of treatment, which may account for a survival benefit in the trial.

In previous researches, it was explained that the activity of chemo-immunotherapy is mainly depends on the presence of an efficient host’s immune response. Cytotoxic drugs were able to induce immunogenic cell death, autophagy and antigen remodeling. In turn, immunological danger signals may empower an efficient tumor-specific immune response (31, 32). In subgroup analysis, we found that eosinophil count in the blood elevated three times higher than baseline level predicted a longer survival. But it is a pity that we did not investigate the underlying mechanisms due to the insufficient study design. The increase of eosinophils in cancer patients has been known for over decades (33). To our knowledge, tumor-infiltrating eosinophils was firstly described in human gastric cancers in the 1980s. The infiltrating of eosinophils suggests a good prognostic value for prolonged survival (30). Eosinophils exert anti-tumor effects *via* direct and indirect mechanisms (34). Eosinophils have been reported to infiltrate multiple tumors, either as an integral part of the tumor microenvironment or in response to various therapeutic strategies. An antitumor role for eosinophils has been demonstrated in various *in vitro* studies. Eosinophil recruitment, prolonged survival and degranulation have been demonstrated in both human and mouse models (35). The above literatures confirm the phenomenon we observed in this study. Lack of randomization restricts our ability to explore the implicated mechanisms, and further studies are needed.

This study has some limitations. Lack of randomization in a single-arm trial restricts our ability to assess the specific role of AGIG. Insufficient sample size limits the accuracy and authenticity of the results. The improvement of the benefit must be considered hypothesis generating. Given the favorable safety profile and the encouraging antitumor activity of the AGIG regimen, validation by a larger randomized trial is necessary.

In conclusion, the AGIG regimen appears more active and safe than the standard AG chemotherapy. To our knowledge, the study demonstrates the antitumor efficacy of a chemo-immunomodulatory strategy in treating advanced PC sufferers for the first time. These results open a new research area for the treatment of pancreatic cancer by combinatory approaches of cytotoxic chemotherapy and immune modulators. Further investigation is warranted.

## Conclusion

The AGIG Chemo-immunotherapy has presented encouraging ORR, DCR, OS, and manageable toxicities as first-line treatment option for advanced PC sufferers. This regimen may be a reliable option for patients with preserved performance status. The improvement of treatment efficiency may result from the activation of non-specific immune response.

## Data Availability Statement

The original contributions presented in the study are included in the article/supplementary material. Further inquiries can be directed to the corresponding authors.

## Ethics Statement

The studies involving human participants were reviewed and approved by the Ethics Committee of Drum Tower Hospital. The patients/participants provided their written informed consent to participate in this study. Written informed consent was obtained from the individual(s) for the publication of any potentially identifiable images or data included in this article.

## Author Contributions

WD has seen the original study data, reviewed the analysis of the data, approved the final manuscript, and is the author responsible for archiving the study files. XQ and CL helped analyzed the data. ZZ, HS, and WK reviewed the analysis of the data and approved the final manuscript. JD and BL helped design the study, analyze the data, and write the manuscript. All authors contributed to the article and approved the submitted version.

## Funding

The study was supported by Chen Xiao-Ping Foundation for the Development of Science an Technology of Hubei Province (No. CXPJJH11900001-2019101). The Affiliated Nanjing Drum Tower Hospital of Nanjing University Medical School sponsored this study.

## Conflict of Interest

The authors declare that the research was conducted in the absence of any commercial or financial relationships that could be construed as a potential conflict of interest.

## Publisher’s Note

All claims expressed in this article are solely those of the authors and do not necessarily represent those of their affiliated organizations, or those of the publisher, the editors and the reviewers. Any product that may be evaluated in this article, or claim that may be made by its manufacturer, is not guaranteed or endorsed by the publisher.
